# Predicting mortality with biomarkers: a population-based prospective cohort study for elderly Costa Ricans

**DOI:** 10.1186/1478-7954-10-11

**Published:** 2012-06-13

**Authors:** Luis Rosero-Bixby, William H Dow

**Affiliations:** 1 Universidad de Costa Rica, Centro Centroamericano de Población, San José, 2060, Costa Rica; 2 University of California at Berkeley, School of Public Health, 239 University Hall, #7360, Berkeley, CA, 94720-7360, USA

**Keywords:** Mortality, Biomarkers, Costa Rica, Aging, Cardiovascular mortality, Death risk factors

## Abstract

**Background:**

Little is known about adult health and mortality relationships outside high-income nations, partly because few datasets have contained biomarker data in representative populations. Our objective is to determine the prognostic value of biomarkers with respect to total and cardiovascular mortality in an elderly population of a middle-income country, as well as the extent to which they mediate the effects of age and sex on mortality.

**Methods:**

This is a prospective population-based study in a nationally representative sample of elderly Costa Ricans. Baseline interviews occurred mostly in 2005 and mortality follow-up went through December 2010. Sample size after excluding observations with missing values: 2,313 individuals and 564 deaths. Main outcome: prospective death rate ratios for 22 baseline biomarkers, which were estimated with hazard regression models.

**Results:**

Biomarkers significantly predict future death above and beyond demographic and self-reported health conditions. The studied biomarkers account for almost half of the effect of age on mortality. However, the sex gap in mortality became several times wider after controlling for biomarkers. The most powerful predictors were simple physical tests: handgrip strength, pulmonary peak flow, and walking speed. Three blood tests also predicted prospective mortality: C-reactive protein (CRP), glycated hemoglobin (HbA1c), and dehydroepiandrosterone sulfate (DHEAS). Strikingly, high blood pressure (BP) and high total cholesterol showed little or no predictive power. Anthropometric measures also failed to show significant mortality effects.

**Conclusions:**

This study adds to the growing evidence that blood markers for CRP, HbA1c, and DHEAS, along with organ-specific functional reserve indicators (handgrip, walking speed, and pulmonary peak flow), are valuable tools for identifying vulnerable elderly. The results also highlight the need to better understand an anomaly noted previously in other settings: despite the continued medical focus on drugs for BP and cholesterol, high levels of BP and cholesterol have little predictive value of mortality in this elderly population.

## Introduction

The study of risk factors of death is central to health metrics [1]. The effects of age and sex are routinely considered in studies of mortality. Marital status, smoking, and obesity are also considered in many mortality studies as shown in a recent systematic review of the literature [2]. Biomarkers are objective physical or biologic measures of health conditions. The availability of information about biomarkers in recent population surveys, mostly on elderly people, has opened the possibility of including biomarkers in population health metrics and in the study of mortality determinants [3]. Biomarkers are studied for their own importance, as well as proximate factors that may help to understand the mechanisms of action of distal factors such as education [4] and to understand the senescence process and the advantage of women in life expectancy [5].

The study of biomarkers as risk factors of death, especially of cardiovascular death or other severe outcomes such as heart attacks, has a longer tradition in health sciences. A major goal in epidemiology is to identify modifiable risk factors that would allow improvements in population health and life expectancy through changes in behavior or in access to existing drugs or with the development of new ones. In turn, physicians often use risk score systems in their clinical practice to identify high-risk individuals and to provide preventive treatment. The paradigmatic Framingham Risk Scores identified the five well-known coronary risk factors: age, smoking, high blood pressure, high cholesterol, and diabetes [6]. Numerous studies have validated the prediction equations of Framingham Risk Scores in other groups [7], have updated them with newer information [8], and have reformulated them with new equations and additional factors such as body mass index, waist circumference, and C-reactive protein [9,10]. The great majority of these studies have been based in observations of middle-age adults from rich countries and more men than women. A more recent generation of studies have examined the mortality predictive value of markers of inflammation such as C-reactive protein, interleukin-6, and fibrinogen [11] and markers of neuroendocrine function including adrenalin, cortisol, and dehydroepiandrosterone [12]. Several studies have shown that biomarkers do improve prediction of mortality using Receiver Operating Characteristic (ROC) curves [13,14], although some also question the performance and added value of traditional blood tests and medical examination above and beyond just age [15].

In spite of the vast literature about risk factors of death, very little is known about health and mortality relationships in adults in the *less developed* regions of the world. This void occurs in part because until recently few data sets have contained the necessary biomarker data in representative populations. Most of the prior mortality research in developing countries has concentrated on children and in communicable diseases, ignoring the current predominant chronic health conditions in the developing world [16]. There is thus a concern about whether traditional risk factors of adult mortality identified with data from middle-aged individuals from rich countries will hold among elderly individuals in developing countries.

The main objective of this study is to determine the prognostic value of biomarkers with respect to total and cardiovascular mortality in an elderly population of a middle-income country, as well as the extent to which they mediate the effects of age and sex on mortality. It intends to determine the effect pattern (positive/negative, linear/nonlinear/U-shaped) of 22 biomarkers, measured at a baseline, on prospective death in a period of about five years. It also aims to test whether traditional coronary risk factors identified in middle aged people from rich countries are also the major factors for elderly Costa Ricans, as well as the association with mortality of newly developed biomarkers of inflammation and neuroendocrine function. Using ROC curves the study intends to establish the added value of the biomarkers to predict further mortality above and beyond self-reported health behaviors and health status at the baseline.

The context for this study is a small, ethnically and socially homogeneous country with a high degree of cohesion and considerable social capital. The 4.5 million Costa Ricans have the second highest life expectancy in the continent (Canada has the highest), higher than richer countries like the US, Chile, or Brazil. Its public health insurance system is almost universal, and its network of primary health care outlets have been quite cost effective at improving the health of disadvantaged groups and erasing many health inequalities [17]. Given that elderly Costa Ricans, particularly males, have been singled out as one of the national populations with among the lowest elderly mortality in the world [18], this study may contribute to understanding some of the proximate determinants of this exceptionally good health. Moreover, the Costa Rican region of Nicoya has been identified as one of a handful of places with exceptional longevity in the world (others are the islands of Sardinia in Italy and Okinawa in Japan), called “blue zones” [19]. This study will check if Nicoya has indeed lower mortality than the already low Costa Rican mortality and the extent to which the Nicoya advantage is mediated by the studied biomarkers.

## Methods

### Ethics statement

This observational study received ethical approval from the *Scientific and Ethical Committee* of the University of Costa Rica in session 63 of March 17, 2004. Each participant and two witnesses signed an informed consent form. The de-identified CRELES databases are publicly available at the National Archive of Computerized Data on Aging (NACDA) of the University of Michigan: http://www.icpsr.umich.edu/icpsrweb/NACDA/studies/26681.

### The CRELES data

We used data from the Costa Rican Study on Longevity and Healthy Aging, known by its Spanish language acronym CRELES. This is a longitudinal study of a nationally representative sample of adults born before 1945 (aged 60 years and over in 2005) residing in Costa Rica, with oversampling of the oldest old. For a nested subsample of about 2,900 respondents, an in-depth longitudinal survey that included biomarkers was carried out, with follow-ups through December 31, 2010 to establish survival. The baseline information on biomarkers and other health conditions was obtained from the first wave of interviews, conducted from November 2004 to September 2006.

The original sample was randomly selected from the 2000 census database after stratification by five-year age groups. Sampling fractions ranged from 1.1% among those born in 1941 to 1945 to 100% for those born in 1900 or earlier. For the in-depth longitudinal survey, the CRELES took a systematic subsample of 60 “health areas” (out of 102 existing in the country), covering 59% of the Costa Rican territory. The baseline interview yielded a response rate of 85% from the located survivors. Among those interviewed, 95% provided a blood sample, 92% collected overnight urine, and 91% had all anthropometric measures. Twenty-four percent of the participants required a proxy to answer the questionnaire. We also included a complementary 100% sample of 91 quasicentenarians (aged 95 years or more) from the area of Nicoya, which was added to the original CRELES sample to increase the statistical power in studies of the exceptional longevity in that area. Sampling weighting factors correct oversampling and differential response rates by age, education, and region.

All the data and blood and urine specimens were collected at the participants’ homes. After answering a 90-minute questionnaire (including mobility tests and two blood pressure measurements), the participants were also instructed to collect overnight urine. Early the next day, fasting blood samples were collected by venipuncture: one EDTA tube of whole blood and two serum-separating tubes with a clot activator. Details about storage of specimens and laboratory procedures are posted at the NACDA web site with documentation of CRELES.

The CRELES field team was trained in a two-week initial course that included standardized anthropometric measures. The blood and urine specimens were analyzed in Costa Rican laboratories certified by the National Reference Centre of Clinical Chemistry, an agency under the Ministry of Health. In addition to routine internal reliability tests conducted in the laboratories, we performed reliability analyses in batches of 20 to 40 specimens that were reanalyzed for each biomarker in a different laboratory. The results of these reliability analyses, as well as some adjustments introduced to standardize measures across the laboratories, have been reported elsewhere [20].

### The dependent variable: death

The CRELES follow up retrieved 813 deaths (564 in individuals with no missing variables) in two ways: (1) through the computer records in the national death registry up to December 31, 2010, and (2) during the second and third waves of home visits. The computer follow-up used the unique identification number (the *cédula*) that all Costa Ricans have. Record linkage with the vital statistics databases, provided by the National Statistics and Census Institute (INEC), allowed us to identify the basic cause of death for 96% of the deceased. Cardiovascular (CV) deaths (codes I001–I999 of the 10^th^ International Classification of Diseases) accounted for 35%.

### Biomarkers studied and their definitions

We define biomarkers as those health indicators objectively measured by well-established laboratory assays on body specimens (blood and urine in this study) or by anthropometrics and other standard devices, such as dynamometers, sphygmomanometers, chronometers, or peak flow meters. Biomarkers are thus objectively measured features of the body, in contrast to the health conditions assessed subjectively by questions and self-reports or by clinical examinations.

The study considered 22 biomarkers grouped in seven dimensions of health. Within each group, some biomarkers may be complementary, while others may be redundant. We tested these issues with the data.

#### Group 1. Metabolic indicators

· Glycosylated hemoglobin (HbA1c), chronic indicator of blood sugar.

· Fasting glucose, indicator of the level of sugar at the moment blood was drawn.

#### Group 2. CV biomarkers

· Diastolic blood pressure (BP), average of two measures taken during the main interview with a digital sphygmomanometer.

· Systolic BP, average of two measures with a digital sphygmomanometer.

#### Group 3. Metabolic-lipids

· Triglycerides in fasting serum.

· Total cholesterol in fasting serum.

· High-density lipoprotein (HDL), or good cholesterol, in fasting serum.

· Total/HDL cholesterol ratio (the two previous markers).

· Low-density lipoprotein (LDL), or bad cholesterol (determined with formula from total cholesterol).

#### Group 4. Stress hormones

· Urinary cortisol, overnight activity in the hypothalamic-pituitary-adrenal (HPA) axis in response to stressors.

· Dehydroepiandrosterone sulfate (DHEAS), an antagonist to HPA activity.

· Epinephrine in overnight urine, an indicator of neuroendocrine functioning.

· Norepinephrine in overnight urine, an indicator of neuroendocrine functioning.

#### Group 5. Inflammation, immune system

· C-reactive protein (CRP).

#### Group 6. Organ-specific functional reserve

· Creatinine clearance in overnight urine as an indicator of kidney reserve.

· Handgrip strength measured with a dynamometer (Creative Health Products Inc., model T-18) as an indicator of arm muscular functioning (we took the higher value of two measurements).

· Walking distance in 10 seconds as an indicator of leg muscular function, derived from the chronometer time required to walk 3 meters, imputing zero distance to disabled participants.

· Pulmonary peak flow, an indicator of pulmonary functioning (we took the highest value of the three measurements obtained with a Mini-Wright meter).

#### Group 7. Nutrition, body shape

· Knee height, an indicator of height that is free from the effects of aging.

· Waist circumference, an indicator of central obesity.

· Body mass index (BMI), an indicator of weight relative to height.

· Waist/hip ratio, an indicator of body shape.

### Control variables

Taking medications that affect some biomarkers:

· High BP medicine.

· Cholesterol-lowering medicine.

· Diabetes medicine.

### Demographic control variables

Sex, age, and residence in the Nicoya region. All the analyses on death risk were controlled for these variables and an interaction between age and sex.

### Baseline health control variables

In some analyses, we considered the following five indicators (we discarded several other indicators that did not show meaningful associations in multiple regressions on mortality, including other categories and indicators of self-rated health, cognitive disability, past history of smoking, indicators of access to health services, and health preventive behavior):

· Normalized disability scale computed with information about help needed in 14 activities of daily living (ADLs).

· Cancer diagnoses reported in the interview.

· Current smoker.

· Self-report of involuntary weight loss of 5 kg or more in the last six months.

· Self-rated health reported as “bad” (the lowest of the five categories).

### Statistical analysis

We normalized all the biomarkers into variables with a mean of zero and a standard deviation (SD) of one. In this way, all their effects on mortality were on the same scale: as the effect of increasing the biomarker in one SD.

We modeled death with parametric proportional hazard models, assuming a Gompertz distribution, which is known to describe well human mortality at adult ages [21]. The proportional assumption was removed by including interactions with age, which was also the survival-time variable. Gompertz’s assumption of linearity in the logarithms of the death rates was examined and modified accordingly by including quadratic terms of the biomarkers in the models. We also allowed for differential effects by sex by including sex interaction variables.

The data were organized as a survival-time dataset, with the date of the 60^th^ birthday as the origin (the birthdates in the CRELES were not self-reported, but taken from official documents, avoiding the problem of age exaggeration that is so pervasive among very old people and which negatively biases the mortality estimates in studies of very old ages). The date of the first interview was the entry point into observation. The exit date was the death date or December 31, 2010 for censored observations of survivors. In the analysis of CV mortality, deaths by other causes are also censored observations on the date of death. To model age properly, we split the time observed in each individual into one-year age segments; in this way, the 2,900 participants in the CRELES became 14,100 observational segments.

We used the STATA-11 software to estimate the death rates and hazard regression models. All standard errors presented are robust estimates. We did not use sampling weights to estimate the regression models, because the models already included the variables that defined sampling weights (age, sex, and Nicoya area).

Following recent literature [13,22], we calculated the ROC curves corresponding to the regression models predicting mortality. ROC curves show the accuracy of a binary classifier (our regression models) to discriminate the true outcomes (deaths). An optimal classifier would be one with 100% sensitivity (all deaths are classified as deaths) and zero false positives among survivors (1 - specificity); i.e., no survivor is classified as a death. The ROC curve shows the tradeoffs between sensitivity and specificity. A random classifier (e.g., flipping a coin) with no discriminatory power will follow a 45-degree line. The area between the ROC curve and the no-discrimination line is the Gini coefficient, broadly used to measure the inequality in a distribution. A Gini coefficient of 1.0 corresponds to that optimal classifier that renders 100% sensitivity and zero false positives.

## Results

The mean age of the individuals in the sample was 79.7 years (10.4 SD) at the entry point (73.3 after correction for sampling weights). Because of oversampling of the oldest old, we had more than 1,300 (46%) individuals aged 80 years or more, which is an unusually large sample size for such old ages. The weighted proportion of women in the sample was 53%. Twelve percent of the participants were in the Nicoya area (8% after weighting). The weighted mean values of the baseline health variables were 2.2 (SD = 3.0) disabilities out of 14 per person, 6% with cancer diagnoses, 10% current smokers, 9% with involuntary weight loss, and 8% reporting bad self-rated health.

Table 1 shows the descriptive statistics for each of the 22 biomarkers. The outliers were excluded from these statistics and in all the analyses as indicated at the table’s footnote. For most of the biomarkers, we had a sample size of about 2,700 observations. The sample sizes were substantially smaller for biomarkers from urine specimens, since we have to discard all the laboratory results for epinephrine and norepinephrine conducted in stored urine specimens, which had degraded. We use only results from fresh specimens, which passed our reliability tests. The table includes information for the subsequent interpretation of the regression results for the normalized biomarkers: the mean and SD used to normalize them, and the proportion of observations at more than 1 SD from the mean. If a biomarker followed a normal distribution, about 16% would be beyond 1 SD and 50% above or below the mean. The distribution by waist circumference was the closest to normal.

**Table 1:** Descriptive statistics of the 22 biomarkers investigated in the study

				**Proportion**			
**Biomarker**	**Units**	**Mean**	**SD**	**≤1SD**	**< mean**	**≥ 1SD**	**N**
*Metabolic hormones*							
Glycosylated hemoglobin (HbA1c)	percent	5.76	1.13	0.01	0.69	0.09	2704
Fasting glucose	mg/dl	110.64	45.39	0.01	0.70	0.09	2748
*CV biomarkers*							
Diastolic blood pressure	mmHg	83.66	12.10	0.15	0.52	0.15	2883
Systolic blood pressure	mmHg	144.00	23.18	0.14	0.55	0.15	2883
*Metabolic – lipids*							
Triglycerides	mg/dl	162.84	85.27	0.10	0.62	0.13	2739
Total cholesterol	mg/dl	215.54	49.42	0.14	0.54	0.15	2746
HDL cholesterol	mg/dl	44.24	13.12	0.14	0.56	0.14	2743
Total/HDL cholesterol ratio	ratio	5.18	1.60	0.15	0.53	0.14	2743
LDL cholesterol	mg/dl	138.48	40.73	0.15	0.54	0.15	2581
*Stress hormones*							
Urinary cortisol	μg/g	26.22	24.99	0.00	0.66	0.09	2249
DHEAS	μg/dl	54.06	41.72	0.10	0.62	0.15	2706
Epinephrine	μg/g	7.41	10.95	0.00	0.69	0.07	1581
Norepinephrine	μg/g	37.63	32.21	0.01	0.65	0.09	1631
*Inflammation, immune system*							
CRP	mg/l	5.61	6.69	0.00	0.73	0.08	2677
*Organ-specific functional reserve*							
Creatinine clearance	mg/min	74.74	30.16	0.14	0.54	0.14	2401
Handgrip strength	kg	26.89	9.08	0.15	0.54	0.18	2595
Distance in 10 seconds	meters	5.51	2.52	0.13	0.46	0.12	2794
Pulmonary peak flow	l/min	304.66	118.65	0.15	0.57	0.17	2635
*Nutrition, body size*							
Knee height	cm	49.41	3.35	0.15	0.52	0.16	2788
Waist circumference	cm	93.88	12.37	0.15	0.51	0.14	2699
BMI	kg/m2	26.87	5.25	0.12	0.50	0.12	2789
Waist/hip ratio	ratio	0.948	0.077	0.14	0.49	0.13	2626

The following outlier observations were dropped: triglycerides: 6 observations >700 mg/dl; LDL-C: 4 obs. >300 mg/dl; cortisol: 7 obs. >400 μg/g; DHEAS: 4 obs. >300 μg/g; epinephrine: 2 obs. >150 μg/g; norepinephrine: 2 obs. >600 μg/g; CRP: 4 obs. >80 mg/l; creatinine: 3 obs. >250 mg/min; and waist circumference: 5 obs < 55 cm.

Statistics computed using sampling weights.

Before using the mortality data from the CRELES, a validity check was necessary. The age-specific death rates from the CRELES are congruent with the series for the entire Costa Rican population from the official life tables for the period 2000 to 2005 [23], suggesting no biases in the sample or errors in the identification of deaths among the participants. Furthermore, both the CRELES and national death rates increase in logarithms almost linearly with age, which makes it appropriate to fit them with a Gompertz function, which also fits CV death rates well (see Additional file 1).

Although prior research suggests that the Costa Rican death registry is complete [18], the CRELES panel is a golden opportunity to assess the registry’s integrity. We were able to successfully match in the registry 99% of deaths found in the field in the second and third waves of interviews: only eight out of 566 deaths retrieved from the field were not found in the registry, accounting for a possible underregistration rate of 1.4% (95% CI: 0.6–2.8). Moreover, as it was possible that some of these eight missing deaths might be registered at some point in the future or might have already been registered with wrong identification, one can safely conclude that the Costa Rican death registry is indeed 100% complete for ages 60 and over. In contrast, about 10% of the deaths in the registry were not found in the field, appearing in the second and third waves as losses of follow-up, which suggests caution in relying exclusively on fieldwork to identify deaths in this or other longitudinal studies.

In a first approach to our research problem we start looking at just the crude effects of each isolated biomarker on mortality, controlling only for demographic variables (age, sex, their interaction, and Nicoya). To estimate them we used a separate Gompertz regression model for each biomarker (Table 2). The purpose of this simplistic approach is to check the direction and size of the effects, as well as to test the existence of nonlinear relationships (with a quadratic term) and whether the effects differ by age or sex as informed by the corresponding interaction terms. As mentioned, the effects, measured as rate ratios (RR), are the proportional changes in the death rate when the biomarker increases by 1 SD.

**Table 2:** Crude death rate ratios of normalized biomarkers estimated with an independent hazard regression model for each biomarker (effect of 1 SD on the death RR)

	**Main**	**Square**	**Age 80+**	**Male**
*Metabolic hormones*				
Glycosylated hemoglobin (HbA1c)	1.42**	0.99	0.89	0.86+
Fasting glucose	1.37**	1.00	0.84+	0.81*
*CV biomarkers*				
Diastolic blood pressure	0.95	1.06**	0.92	1.06
Systolic blood pressure	0.88+	1.07**	0.89	1.11+
*Metabolic – lipids*				
Triglycerides	0.90	1.05**	0.88	1.04
Total cholesterol	0.79**	1.07**	1.09	1.02
HDL cholesterol	0.84*	1.05*	1.15	1.00
Total/HDL cholesterol ratio	0.99	1.02**	0.89	1.01
LDL cholesterol	0.80*	1.04+	1.08	1.02
*Stress hormones*				
Urinary cortisol	1.00	0.99	1.15	1.12+
DHEAS	0.90	1.08**	0.93	0.87
Epinephrine	1.16	1.00	0.88	0.85
Norepinephrine	0.98	1.01	1.06	1.09
*Inflammation, immune system*				
CRP	1.42**	0.97*	0.93	0.99
*Organ-specific functional reserve*				
Creatinine clearance	0.72**	1.10**	1.21+	0.97
Handgrip strength	0.41**	0.98	0.98	1.69**
Distance in 10 seconds	0.56**	1.01	1.16+	1.08
Pulmonary peak flow	0.47**	0.97	0.97	1.39*
*Nutrition, body size*				
Knee height	0.92	0.97	0.98	1.14
Waist circumference	1.08	1.06**	0.85+	1.02
BMI	1.04	1.02**	0.77**	0.98
Waist/hip ratio	1.16+	0.99	0.85+	0.99

Significance assessed with robust estimates of standard errors: + means significant at *p < 0.10; at **p < 0.05; and at *p* < 0.01.

All regression models also include control variables for sex, age (continuous), and Nicoya.

The following example illustrates how to read the estimated effects in Table 2. A female aged 70 years with a C-reactive protein level that is one standard deviation above the mean has an annual death risk of 1.38 (1.42 * 0.97) compared to a woman with similar characteristics and a CRP level equal to the mean (which is 5.6 mg/l). If the person in this example had a CRP level 2-SD above the mean, her relative death risk would be 1.81 (1.42 ^ 2 * 0.97 ^ 4); i.e., an 81% higher risk of dying in the coming year. If the person of the example were 85 years old, his death RR would be 1.69 instead. If the person were a man, instead of a woman, the estimates would be essentially the same given that the effect of being male is very close to 1.

The biomarkers in the group “organ-specific functional reserve” showed the strongest effects on mortality. One SD increase in these biomarkers reduced death risk by approximately half. There were also strong effects on mortality from CRP and HbA1c biomarkers in the order of a 40% increase in mortality.

In the group of stress hormones, the only biomarker that significantly predicted mortality in this sample was DHEAS.

Body mass and shape biomarkers showed weak effects, which differed by age. It must be noted that among individuals older than 80 years, a larger BMI or waist circumference reduced the risk of dying.

**Figure 1:**
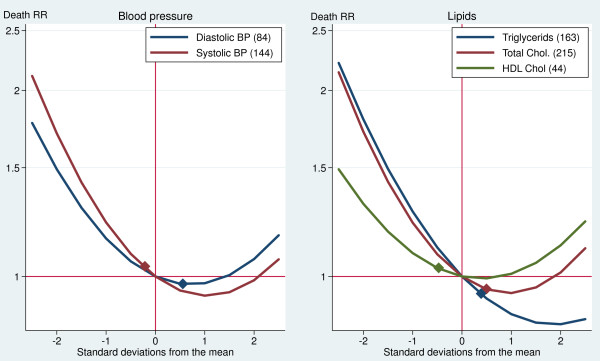
**Crude effects of blood pressure and lipid biomarkers on death rate ratios (Costa Rican males aged 80+).** Notes: The numbers within parentheses in the legends are the mean values. The diamonds indicate the cutoff levels of metabolic risk used in clinical practice. Although these curves are for males aged 80+ years, the curves for females and ages 60 to 79 years are similar, given that the interactions with age and sex in Table 2 are small and nonsignificant.

BP and lipid profile, which are biomarkers broadly used in clinical practice, showed complex curvilinear effects. Figure 1 translates the coefficients in Table 2 into curves showing the effect on mortality of the full range of these biomarker levels. The curves show that extreme levels, both low and high, increase the risk of dying. They also suggest that low levels of BP and cholesterol are more risky than high levels in this population. At −2 SD, the death RRs ranged between 1.4 and 2.1 when compared with the mean values. On the other extreme of the curves, the death RRs associated with 2 SD above the mean ranged between 0.8 and 1.2.

The U-shape of the curves in Figure 1 indicates that there is an optimal level in the biomarker that minimizes the death rate: a diastolic/systolic BP of 176/92 in females and 154/85 in males at the ages of 60 to 79 years, and of 186/96 in females and 164/89 in males at the ages of 80 or more years (figures computed from the estimates in Table 2). The optimal levels were about the same as the sample’s average for diastolic BP (84 mmHg) and HDL cholesterol (44 mg/dl). In contrast, for systolic BP, total cholesterol, and triglycerides, the optimal level was to the right (at higher levels) of the mean. The diamonds in Figure 1 show the cutoff levels recommended by the American Heart Association to define metabolic risk (>140 mmHg for systolic BP, >90 mmHg for diastolic BP, >250 mg/dl for total cholesterol, >200 mg/dl for triglycerides, and <40 mg/dl for HDL). Individuals above these levels are considered at risk, except for HDL, which identifies at-risk individuals as those below the cutoff. If these curves are true causal relationships, some conventional medical interventions would be harmful in this population: reducing the levels of systolic BP, total cholesterol, and, especially, triglycerides in individuals slightly above the cutoffs would increase their risk of dying.

To what extent are the biomarkers independent from each other? Which biomarkers overlap each other? The correlation coefficients between pairs of biomarkers allow a quick examination of this point ( Additional file 2 summarizes the matrix of correlation coefficients showing those that are 0.25 or higher). As expected, there are several colinearities between biomarkers within the same group: total and LDL cholesterol (r = 0.95), systolic and diastolic BP (r = 0.72), waist circumference and BMI (r = 0.76), and handgrip strength and spirometry (r = 0.65). However, no meaningful correlations exist between biomarkers from different groups. The exceptions are DHEAS, grip strength, and knee height, although their intercorrelations seem to be a spurious result of their association with sex.

Interestingly, the four biomarkers in the group of organ-specific functional reserve are the only ones associated with age ( Additional file 2). Therefore, creatinine clearance, handgrip strength, rapid walking, and pulmonary peak flow might be taken by themselves as indicators of aging. That is not the case for other biomarkers such as blood pressure and lipid levels, which are not clearly associated to the age of the individuals.

An important research issue is whether some baseline health conditions determine in part the biomarkers’ level, which would confound their crude effects on mortality presented in Table 2. For example, individuals with low levels of triglycerides could also be undernourished or could even suffer from serious diseases, such as cancer, and thus their high death rates could be a consequence of those conditions and not of the low levels of triglycerides *per se*. To address these issues, as well as to exclude biomarkers that are redundant, we need to estimate controlled effects on mortality in multivariate regression models. We could estimate a single regression model that includes all the biomarkers and baseline health factors in the equation. This, however, would not be an efficient model. By including explanatory variables that we know beforehand to be redundant to each other, or by including variables that we know have no effect on mortality or have many missing values, we would lose statistical power and degrees of freedom, and thus obtain less reliable estimates. To reduce these problems, we decided to exclude from multivariate analyses those biomarkers with no crude effects or with substantially less than 2,700 observations (stress hormones, except DHEAS, creatinine, and knee height) from the analysis. We also dropped redundant biomarkers (fasting glucose, diastolic BP, triglycerides, LDL cholesterol, and waist/hip ratio), which we identified as those that do not add significant explanation for the death rates in stepwise regressions within each group of biomarkers. In addition, we kept in the model only those biomarkers’ quadratic terms and interactions with age and sex that were significant.

Table 3 shows the multivariate estimates for the 12 remaining biomarkers. In addition to the basic demographic model, the table shows three sets of estimates: (1) partial models that include demographic variables and biomarkers of the same group only, (2) a full model that estimates the effect of a biomarker net of all the other biomarkers and the demographic controls, and (3) a second full model that additionally controls for the potentially confounding effects of baseline health variables. To obtain comparable results from the different models, we excluded individuals with missing values in any of the variables. All the models were thus estimated for the same sample of 2,313 individuals and 564 deaths.

**Table 3:** Death rate ratios of normalized biomarkers estimated with several specifications of hazard regression models (effect of 1 SD on the death rate)

**Biomarkers and control variables**	**Demo-graphic model**	**Partial models**	**Full model 1**	**Full model 2**
*Metabolic hormones*				
Glycosylated hemoglobin (HbA1c)		1.23**	1.23**	1.24**
*Cardiovascular biomarkers*				
Systolic blood pressure (SBP)		0.85**	0.89*	0.90+
quared SBP		1.05*	1.03	1.03
SBP * Male			1.15+	1.13	1.15+
*Metabolic – lipids*					
Total cholesterol		0.73**	0.83**	0.85*	
Squared total chol.		1.08**	1.04+	1.03	
HDL cholesterol		1.26**	1.19*	1.16*	
Total/HDL cholesterol ratio		1.24**	1.19**	1.16*	
*Stress hormones*					
DHEAS		0.79**	0.83**	0.84*	
Squared DHEAS		1.08**	1.05*	1.04	
*Inflammation, immune system*					
CRP		1.43**	1.27**	1.24**	
Squared CRP		0.96**	0.97*	0.97+	
*Organ-specific functional reserve*					
Handgrip strength		0.55**	0.59**	0.65**	
Handgrip * Male		1.62**	1.60**	1.53**	
Distance in 10 seconds		0.75**	0.79**	0.90+	
Pulmonary peak flow		0.71**	0.72**	0.73**	
*Nutrition, body size*					
Waist circumference		1.04	0.99	1.00	
Squared waist circumference		1.06**	1.06*	1.06*	
BMI * age 80+		0.84*	0.94	0.92	
*Baseline health controls*					
Normalized disability scale		1.59**		1.27**	
Cancer diagnosis		1.46**		1.51**	
Involuntary weight loss		1.24*		1.17	
Current smoker		1.43+		1.50*	
Bad self-reported health		1.22		1.12	
*Demographic controls*					
Male	1.65*		4.31**	3.76**	
Male * Age	0.99		0.99	0.99	
Nicoya	0.71*		0.66**	0.73*	
Age (1 extra year)	1.09**		1.05**	1.05**	

Significance assessed with robust estimates of standard errors: + means significant at *p < 0.10; at **p < 0.05; and at *p* < 0.01.

Partial models include the biomarkers of the respective group AND demographic controls. *N* = 2,313 individuals, 11,433 observed age segments, and 564 deaths.

There are no considerable changes in the magnitude and significance of the effects of the biomarkers when one moves from the partial to the full models (Table 3), except for the effects of nutrition, which tend to disappear and become nonsignificant. This change could be interpreted to indicate that nutrition (body mass and shape) has no independent effect on mortality in this population. It could also be interpreted to signify that nutrition effects are indirect or mediated by other biomarkers, such as blood sugar or BP, and thus should not be placed in the same equation, but modeled in a multi-equation way.

There are also no considerable differences in the rate ratios when we introduce the control for baseline health in the last column in Table 3. The small decreases in the magnitude of several RRs (movements toward 1.0) can be interpreted as the confounding effects of baseline health conditions on the relationships between the biomarkers and death. Such decreases are not important, and the effects do not change in statistical significance.

Table 3 also answers the question of whether these biomarkers explain sex, age, and other variations in death rates. The 9% increase in mortality per year of age in the demographic-only model drops to 5% after the biomarkers are introduced into the model. Therefore, the biomarkers in the model would explain about half of the senescence effect (more precisely, the *gamma* coefficient of the Gompertz mortality function estimated with no interactions for age or sex falls from 8.21% to 4.11% when the biomarkers are added to the demographic model; i.e., the biomarkers explain 49.9% of the effect of aging on mortality). The effects of disability and self-reported health also drop by half when the biomarkers are entered into the model. In contrast, the higher risks of dying associated with smoking and cancer are found to have no relation with the biomarkers. In addition, the mortality-reducing effect of residing in Nicoya (death RR of 0.71) changed little after controlling for biomarkers.

The death rate for Costa Rican males by age 60 years is 1.65 times higher than that for females (Table 3). This gap increases to 4.3 times when the model introduces the biomarkers, signifying that the sex gap in mortality is not caused by the biomarkers shown in the table. On the contrary, the mystery of the excess mortality of males deepens when one takes into account that women usually show higher risk levels in several biomarkers.

**Figure 2:**
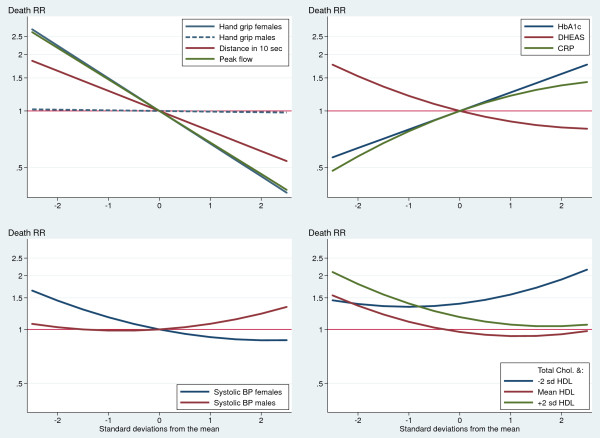
Controlled effects of normalized biomarkers on death rate ratios (elderly Costa Ricans).

Figure 2 summarizes the final effects of the biomarkers on death, as estimated by the model in the last column of Table 3 (nutrition biomarkers are not plotted because they are not significant). The strongest and most straightforward predictors of death are also those basic biomarkers that can be measured inexpensively with a dynamometer, a chronometer, or a peak flow meter, i.e., the group of organ-specific functional reserve biomarkers. For example, the death rate for women at the lowest end (−2 SD) of the handgrip distribution is more than five-fold higher than that for women at the other tail (+2 SD) of the distribution. (The discriminatory power of the handgrip test is, however, nil for men.)

Furthermore, there are three blood tests with clear and consistent predictive power for higher death risks: high levels of CRP and HbA1c and low levels of DHEAS. Individuals at the higher tail of the distribution of CRP or HbA1c were found to have about three times higher death rates than those at the lowest end. The effect of DHEAS was found to be a bit smaller and in the opposite direction: reduced hormonal levels were associated with a two-fold increase in mortality, when the extremes of the distribution are compared.

Systolic BP appeared as a weak predictor of mortality, and it had opposite effects depending on sex. High levels of systolic BP predicted a slightly higher risk of dying among men, but strikingly lower death rates among women.

The estimated effects of total and HDL cholesterol are complex (Figure 2). At low HDL levels, individuals with high total cholesterol were found to be more likely to die. In contrast, at medium or high HDL (the “good” cholesterol) levels, the effect of total cholesterol reversed and strikingly became a protective factor against death.

**Figure 3:**
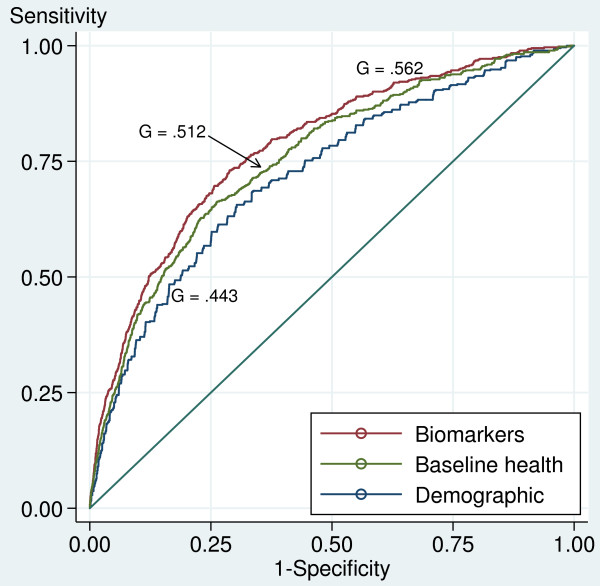
**ROC curves for models predicting death with estimated death hazards.** G = Gini coefficient or proportional discrimination area (between the curve and the no-discrimination line).

What is the joint effect of the biomarkers in predicting mortality in this sample? The ROC curves corresponding to the regression models presented in Table 3 show the accuracy of these models as binary classifiers to discriminate the true outcomes (deaths), as shown in Figure 3. A Gini coefficient of 1.0 corresponds to an optimal classifier that renders 100% sensitivity and zero false positives. Our full regression model with biomarkers, baseline health, and demographic variables demonstrated a Gini of 0.56 as the classifier of death. This model, for example, discriminates death with 85% sensitivity and 46% of false positives at a cutoff level of 0.03 for the predicted death hazards.

The model that includes biomarkers improved the demographics-only model (age, sex, and Nicoya) significantly: the Gini area increased from 0.443 to 0.553, or by 25% (not shown in the Figure 3). The biomarkers also significantly improved the model with baseline health and demographic indicators: the Gini increased from 0.512 to 0.562, or by 10%. However, Figure 3 also puts the results in perspective: the discrimination area added by the biomarkers is relatively small when compared with that achieved by the simple demographic model of age and sex, i.e., the life table.

### Cardiovascular (CV) mortality

**Figure 4:**
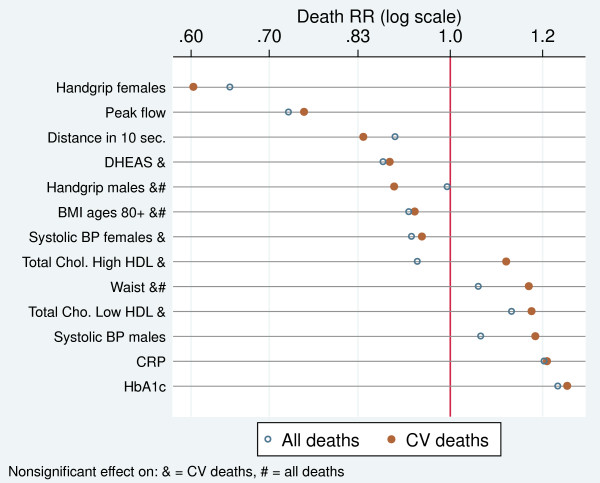
Controlled effects of increasing biomarker levels by 1 SD from the mean values on death rate ratios by all causes and CV diseases.

Many biomarkers in the CRELES are markers of CV risk. We should thus focus on their effect on CV deaths only (with the limitation that statistical power is reduced when we move from 564 all-cause deaths to 213 CV deaths in our final model). We re-estimated the full regression model with baseline health controls presented in Table 3 for CV deaths, with individuals dying from other causes taken as censored observations. Figure 4 compares the death RRs estimated with the model for CV mortality (full, red dots) with those obtained for all-cause mortality (hollow, blue symbols) from Table 3, full model 2. The figure shows the effect of increasing the biomarker from the mean to 1 SD. The dots to the left of the RR = 1 line indicate that these biomarkers protect against death; the dots at the right indicate an elevated risk of death. The biomarkers in the figure are ordered from those more protective to those more risky for CV mortality. At the center of the figure are those neutral in their death risk.

For most of the biomarkers, the effect on CV mortality was found to be larger than that on all-cause deaths. However, because of the diminished statistical power, several of the estimated effects on CV mortality were nonsignificant, even if they were larger than those on all-cause mortality.

The most powerful predictors of CV death were the organ-specific function markers: handgrip (only for females) and peak flow. One SD above the mean in these biomarkers reduced the CV deaths by 40% and 25%, respectively. At the other extreme, three biomarkers significantly increased the CV mortality by about 20%: HbA1c, CRP, and systolic BP in males.

Among older (80+) individuals, higher BMI appeared to be associated with low CV mortality, whereas a larger waist circumference seemed to be related to about 20% higher CV mortality; however, we did not have enough statistical power to establish this effect as significant.

The two traditional biomarkers of CV risk, BP and cholesterol, showed weak and nonsignificant effects on CV mortality, with one exception: a higher risk for males with increased systolic BP.

## Discussion

The biomarkers in this sample demonstrated significant ability to predict future death above and beyond demographics and self-reported baseline health conditions. However, as shown by the ROC curves, the added predictive value of the biomarkers was small when compared with that of two easily observable biological traits: age and sex. This small added value does not mean, however, that biomarkers are not important to understand mortality, but that biomarkers may act more like proximate or mediating factors of the aging process. The examined biomarkers explained half of the effect of age on mortality.

In contrast with age, the sex gap in mortality became several times wider after controlling for the biomarkers. The mystery of the excess mortality of males deepened when one considered that females were the ones with a disadvantageous profile of biomarkers.

The most powerful predictors of future death, especially CV death, were three simple physical tests: handgrip strength for females, pulmonary peak flow, and walking speed. Three blood tests also predicted higher risk of mortality: CRP, HbA1c, and low DHEAS. Strikingly, two groups of biomarkers, broadly accepted in medicine as risk factors – high BP and high total cholesterol (and triglyceride) levels – were found to have little to no ability to predict future death (all-cause or CV) in this dataset, with the exception of a higher CV mortality predicted for males with high systolic BP. Furthermore, anthropometric measures failed to show significant effects when other biomarkers were controlled for.

The studied factors—biomarkers—are objectively measured and validated indicators, as is the endpoint definition: death. In contrast to studies of mortality in elderly populations from developing countries, age misreporting is not an issue because the CRELES uses only well-documented birth dates. Underregistration of deaths is unlikely, because the deaths are identified twice: by follow-up in the field (two visits) and by computer linkage with the death registry. Moreover, the CRELES fieldwork demonstrated the completeness of the Costa Rican death registry. In addition, the external validity of the study is assured by its population-based nationally representative sample. Because of oversampling of the oldest old, there is a good representation of very old individuals as well as higher statistical power. Taking biomarkers as continuous variables (instead as binary risk factors as many epidemiologic studies do) and allowing for curvilinear associations as well as effect modifications by age and sex made it possible to identify complex, nonlinear patterns. The use of a normalized scale for all biomarkers made their effects comparable. The general stability of the biomarkers’ effects on mortality, regardless of whether other demographic and health variables are controlled for, suggests that the estimated effects are robust.

A weakness of the study is its limited statistical power, especially for identifying the effects on CV mortality. The final analysis was based only on the observation of 213 CV deaths, a small sample size that requires large effects to reach statistical significance. Nevertheless, the sample is large when compared with those in key previous studies in the literature from outside developed countries, such as the SEBAS survey carried out in Taiwan [22].

Another potential weakness is the contamination of the sample after the first wave of visits. Although the CRELES did not intervene to modify the health of participants, because of ethical considerations the results of well-established markers of CV risk were communicated to participants with a recommendation of visiting a physician when necessary.

A concern regarding our intent of quantifying the mediating effects of biomarkers in the relationship of age and sex with mortality is that our conclusions are strictly valid only under the assumptions that there is no *confounding* between the intermediate effects (biomarkers) and mortality and that there is no synergism, i.e. that age or sex does not interact with biomarkers to cause mortality [24]. As with most findings from observational studies the estimated associations can only been taken as preliminary assessments of possible causal effects to be checked with better designed studies.

We purposely neither included controls for socioeconomic status in this study nor addressed their relationship with the biomarkers. These are complex issues addressed in other analyses of this dataset [4,17,25]. Clinical use of these biomarkers for identifying high risk individuals is typically done without controlling for SES, so similarly we want to show these straightforward models here. In any case, we checked that our results are not sensitive to the control for education effects by estimating the full model for all-cause and CV deaths. The inclusion in the regression model of the variable years of education and its square did not change the effects of biomarkers on mortality shown in Figure 4.

A problem in studies of elderly individuals is the potential confounding effect of earlier conditions. However, the result that the effects of biomarkers change little when a set of baseline health conditions is controlled for in the models is an encouraging hint of the robustness of the results to those hard-to-measure earlier conditions.

The simultaneous investigation of the effects of 22 biomarkers is both a strength and weakness of the study. It allows for a comprehensive assessment of the extent to which biomarkers can predict mortality or can add to what is known about the determinants of mortality at old ages, as we did using the Gini indicator of the discrimination area under the ROC curve. The downside of this comprehensive approach is that we could not examine each of the relationships in depth, as would have been the case in a typical epidemiologic study of one factor on one outcome.

### Comparisons with other studies

Mortality rates in this sample of older Costa Ricans are similar to that observed in the general population. Costa Rica is known for having mortality rates at adult ages lower than in many rich countries [18]. The life expectancy at age 60 corresponding to the CRELES series of death rates is 23.2 years, which is a half-year higher than that in the US, although it is 1.7 years shorter than that in Japan according to the Human Mortality Database [26].

The Gompertz gamma coefficient of 8.7% estimated here with CRELES data is in the range of the Gompertz “law” that after age 35 human mortality increases by 8% to 14% per year, according to data from rich countries [1]. The CRELES-based figure is, however, at the lower end of this interval, suggesting that senescence among elderly Costa Ricans occurs at a slower pace than in other populations.

When Costa Rican biomarker levels are compared with the published results from the SEBAS study carried out in Taiwan [27], and the MacArthur, NHANES, and HRS studies carried out in the US [4,12,25], elderly Costa Ricans are found to be the worse off in most of their biomarkers, a paradoxical result given the higher life expectancy of adult Costa Ricans. For example, the mean systolic BP was 144 mmHg in Costa Rica, compared with 138 in the US and Taiwan. The prevalence of hypercholesterolemia (>250 mg/dL) in Costa Rica (30% of women and 15% of men) was more than double of that observed in Taiwan and the US [4]. The only biomarkers with healthier levels in Costa Rica were blood sugar, body mass index, and norepinephrine when compared with the US and blood sugar when compared with Taiwan.

This study confirms and expands the findings of the SEBAS study of elderly Taiwanese mortality that prediction is significantly improved by biomarkers and that the standard biomarkers of CV risk have lower predictive value than markers from the neuroendocrine and immune systems [13,22,27]. Given that the SEBAS study was limited in its small sample size (927 subjects and 162 deaths in the most recent analysis), the CRELES provides more precise estimates.

This study confirms consistent inverse associations between the measures of physical capability (such as grip strength and walking speed) and mortality in older populations well documented in the literature [28]. Our data are also consistent with those documenting a positive effect of HbA1c and CRP and an inverse effect of DHEAS on mortality by all causes and CV conditions [29-35]. However, there are not many studies on the mortality effect of these three relatively new biomarkers.

In contrast, there is a large body of literature showing the direct association of high BP and high total cholesterol with mortality. However, the great majority of these studies are for middle-aged individuals in developed countries. The CRELES data analyzed in this study are thus at odds with the paradigm that high BP and cholesterol levels are important mortality risk factors. This is not the first study failing to document this relationship. For example, studies in Hawaii [36], Korea [37], the Netherlands [38], and the US [39] did not find that lowering cholesterol levels reduced mortality in elderly people. A reexamination of the data from the Framingham Heart Study led to the conclusion that the direct association between cholesterol and mortality attenuates and even reverses with age and recommended caution on cholesterol-lowering treatments in elderly people [40]. The SEBAS study among elderly Taiwanese found no evidence that high BP and high cholesterol are associated with up to six-year prospective mortality [13,22].

In general, studies that stratify mortality effects by age find that among older individuals, the effects of these biomarkers are weaker or even disappear. A possibility is that the current massive use of prescription drugs conceals the effect on mortality of hypertension and high cholesterol (among elderly Costa Ricans, 44% were found to take antihypertension drugs and 21% were taking cholesterol-reducing drugs, according to the CRELES). We explored this possibility by estimating regression models that included variables for taking these medicines and their interactions with the biomarker levels. No significant effects of these medicines were found. Taking these medicines was neither a confounder nor a modifier of the effect of the biomarkers. An exception was a higher risk of CV death among individuals taking BP medicine, suggesting a complex relationship that merits an in-depth epidemiologic analysis of the data, which is out of the scope of the present study.

Another explanation is that survivors at old ages are a selected group of individuals among whom the effects of traditional risk factors (usually documented with data for middle-aged individuals) act differently or have no effect. Sorting out this survival-selection effect is not simple and is a pending research task in order to understand health and mortality in elderly populations. Some hints of this explanation may come from looking at the interaction effects of age (80+ compared to 60 to 79 years) and each biomarker shown in Table 2. With the exception of BMI, the effects of biomarkers are generally similar in the two age groups, which suggest that selection effects are unlikely.

Our findings are also in line with reports that in some special populations (individuals with advanced age, heart failure, malignancies, AIDS, on hemodialysis, and so on) associations between biomarkers and mortality are different and even in the opposite direction than in the general population. These situations have been termed “reverse epidemiology.” It is not, however, clear whether this reversal is a real or spurious association caused by other conditions, such as the malnutrition-inflammation complex syndrome [41].

From another perspective, the weak association between traditional CV biomarkers and mortality explains at least in part the paradox that whereas adult Costa Ricans have exceptionally high levels of life expectancy and that their advantage mostly comes from relatively low levels of CV mortality [18,42], they are also at higher metabolic risk with regard to BP and cholesterol, when compared with the elderly in countries such as Taiwan or the US.

## Conclusions

Although this study does not go as far as to establish causal relationships that would immediately translate into clinical practice, its results can be used to identify individuals and populations at high risk of dying, as well as to generate hypotheses about the possible pathways toward higher or lower mortality levels. In addition, these results can also be useful for checking whether the paradigms derived from studies carried out in developed countries, such as the Framingham study [8] hold for a developing nation that is following a different path in its epidemiological and nutritional transitions [17,18].

In this study, organ-specific functional reserve biomarkers (handgrip, walking speed, and pulmonary peak flow), along with CRP, HbA1c, and DHEAS, have been found to be suitable biomarkers for improving the identification of vulnerable individuals in an elderly population of the developing world. There is now growing evidence that these biomarkers may be important in both clinical practice and public health surveillance. These biomarkers are also valuable in surveys assessing health and survival, as well as in understanding the aging process. More research is obviously needed to understand the causal mechanisms underlying these associations. Most of these biomarkers with a high predictive value cannot be taken as causal factors or as a disease, they are just a symptom or an indicator of a health problem.

The lack of evidence backing well-established medical paradigms regarding hypertension and hypercholesterolemia is a striking result that certainly merits further research. The health expenditures for prescription drugs to control these conditions are substantial in Costa Rica and elsewhere, and the value of these therapies for elderly populations and in settings similar to Costa Rica should be better evaluated.

Medicine needs a deeper understanding of the meaning of some biomarkers in elderly populations, as well as outside of the developed country settings, where they have been primarily studied. Given this lack of information, we cannot tell whether the results found for elderly Costa Ricans is a peculiarity of this country, whose adult population has exceptionally high life expectancy, or whether they may be extrapolated to other adult populations in the developing world.

## Abbreviations

ADL: Activities of daily living; BMI: Body mass index; BP: Blood pressure; CI: Confidence interval; CRELES: Costa Rican Study on Longevity and Healthy Aging; CRP: C-reactive protein; CV: Cardiovascular; DHEAS: Dehydroepiandrosterone sulfate; EDTA: Ethylenediaminetetraacetic acid (an anticoagulant); HbA1c: Glycated hemoglobin; HDL: High-density lipoprotein; HPA: Hypothalamic-pituitary-adrenal axis; INEC: National Statistics and Census Institute (Costa Rica); LDL: Low-density lipoprotein; NACDA: National Archive of Computerized Data on Aging; NHANES: National Health and Nutrition Examination Survey; ROC: Receiver Operating Characteristic curves; RR: Rate ratio; SD: Standard deviation; SEBAS: Social Environment and Biomarkers of Aging Study (Taiwan); US: United States of America.

## Competing interest

We declare that we have no competing interests.

## Authors’ contributions

LR-B and WD originated and designed the CRELES study. LR-B directed and organized data and specimen collection, including quality controls of laboratory procedures. LR-B conducted the data analyses and wrote the paper with advice and collaboration of WD. LR-B is guarantor for this paper. Both authors read and approved the final manuscript.

## Supplementary Material

Additional file 1 This figure compares the age-specific mortality rates of the CRELES sample to the national estimates in the official life tables and shows that a Gompertz model fits them well. Click here for file

Additional file 2 This figure is a correlogram showing pair-wise correlation coefficients larger than 0.25 between the 22 biomarkers in the study and age and sex. Click here for file
