# Supplementary material for: Predicting mortality with biomarkers: a population-based prospective cohort study for elderly Costa Ricans
Source: Popul Health Metr. 2012 Jun 13;10:11. doi: 10.1186/1478-7954-10-11 (PMC3507767; doi:10.1186/1478-7954-10-11)
Supplement: Additional file 1 — This figure compares the age-specific mortality rates of the CRELES sample to the national estimates in the official life tables and shows that a Gompertz model fits them well. [file 1478-7954-10-11-S1.pdf]

Additional File 1.

Age-specific death rates (mx). The CRELES and the Costa Rican life table.

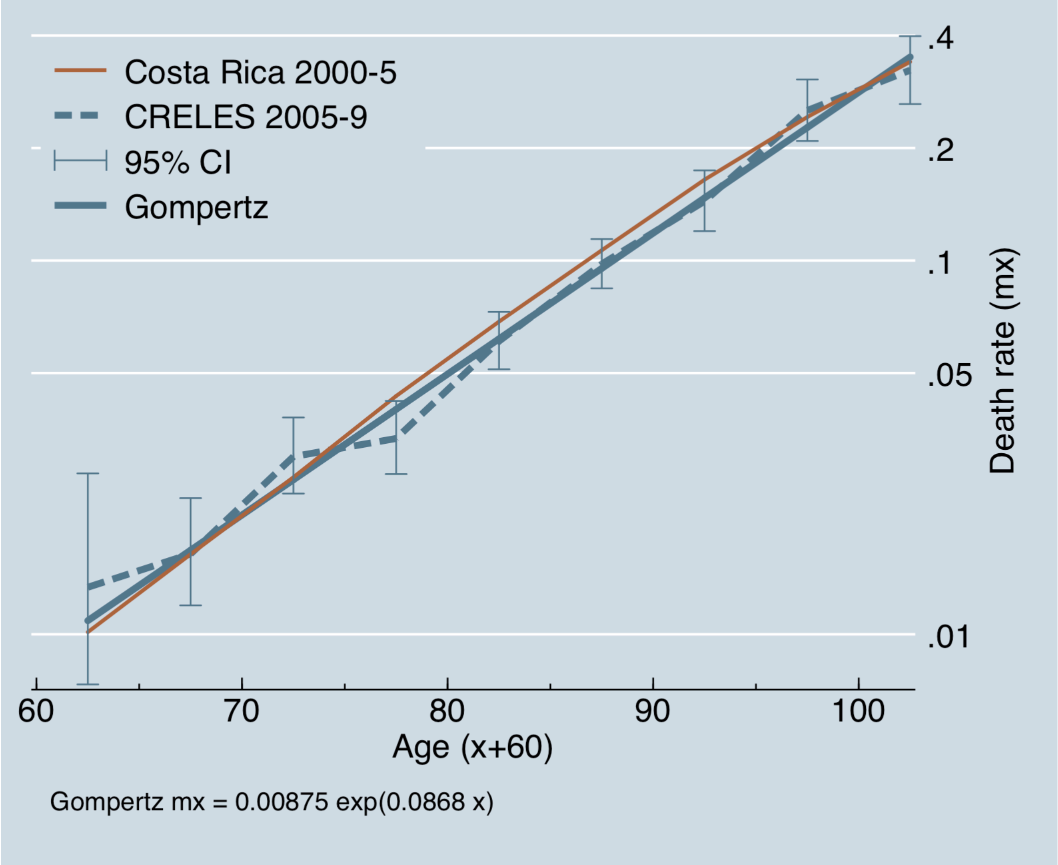

Life expectancy at the age of 60 years is 22.7 in the CR life table and 23.2 in the CRELES sample.
The constant term or intersection (0.0087) of the Gompertz function estimates the death rate at age 60. The "*gamma*" coefficient estimates the rate at which mortality increases with each year of age: 8.7% (95% CI: 8.0–9.4) per year.
For CV deaths only the Gompertz function also fits well the data with intersection at 0.0028 and a gamma coefficient of 8.9%.
