# Supplementary material for: Predicting mortality with biomarkers: a population-based prospective cohort study for elderly Costa Ricans
Source: Popul Health Metr. 2012 Jun 13;10:11. doi: 10.1186/1478-7954-10-11 (PMC3507767; doi:10.1186/1478-7954-10-11)
Supplement: Additional file 2 — This figure is a correlogram showing pair-wise correlation coefficients larger than 0.25 between the 22 biomarkers in the study and age and sex. [file 1478-7954-10-11-S2.pdf]

Additional File 2

Pair wise correlation coefficients between biomarkers.

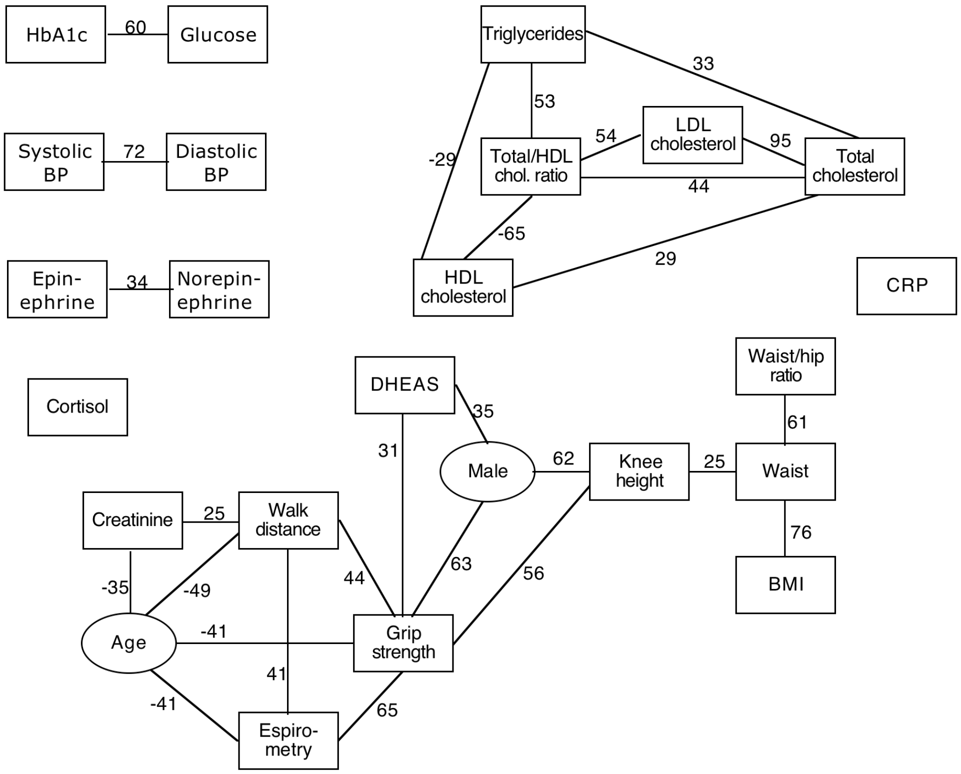

The numbers indicate correlation coefficients in percentages. Only 0.25 or higher coefficients shown.
